# Genome sequencing-based CNV analysis in an infant with concurrent partial trisomies 9p and 12p due to maternal translocation: A case report

**DOI:** 10.1097/MD.0000000000049159

**Published:** 2026-06-05

**Authors:** Zohor Azher, Malak Alsabban, Lujain Akram Ali, Hind Naffadi, Samar Ekram, Rami Obaid

**Affiliations:** a Department of Medical Genetics, Faculty of Medicine, Umm Al-Qura University, Makkah, Saudi Arabia; b Faculty of Medicine, Umm Al-Qura University, Makkah, Saudi Arabia; c Department of Medical Genetics, Faculty of Medicine, Al-Qunfudah Branch, Umm Al-Qura University, Al-Qunfudah, Saudi Arabia.

**Keywords:** copy number variation, dysmorphic infant, genome sequencing-based CNV analysis, partial trisomy 9p, partial trisomy 12p

## Abstract

**Rationale:**

Copy number variants (CNVs) are structural genomic alterations that can lead to a range of genetic disorders by disrupting gene dosage and function. Large duplications affecting multiple genes are known to result in partial trisomies, frequently associated with developmental delay, intellectual disability, and congenital anomalies. These types of CNVs often arise from unbalanced chromosomal translocations, typically inherited from phenotypically normal carriers of balanced rearrangements. This study describes the clinical and cytogenomic findings of a rare case of a dysmorphic infant with concurrent partial trisomies of chromosomes 9p and 12p.

**Patient concerns:**

A female infant presenting with global developmental delay, growth retardation, distinctive craniofacial dysmorphism, and multiple congenital anomalies. Conventional karyotype, along with genome sequencing-based CNV analysis, has been performed. In addition, parental karyotyping has been performed.

**Diagnoses:**

The infant’s karyotype revealed an extra copy of a structurally abnormal chromosome 9. Genome sequencing-based CNV analysis identified 2 pathogenic CNVs: a duplication of chromosome 9p24.3-q21.13 and a second duplication of chromosome 12p13.33-p12.1, indicating 2 partial trisomies of chromosome 9p and 12p. Parental karyotyping revealed a maternally inherited balanced translocation involving chromosomes 9 and 12.

**Interventions and outcomes:**

The patient received supportive care; at 12 months of age, she developed a severe respiratory infection and died.

**Lessons:**

This case contributes to the expanding phenotypic and cytogenomic spectrum of partial trisomies 9p and 12p. We highlight the role of CNV analysis based on genome sequencing in the clinical evaluation of individuals with dysmorphic and malformative conditions. We also emphasize the importance of parental cytogenetic studies in such cases for elucidating inheritance patterns and recurrence risks.

## 1. Introduction

Genomic structural variants (SVs) are a class of genomic alterations characterized by the rearrangement of deoxyribonucleic acid (DNA) segments larger than 50 nucleotides.^[1]^ These variations can significantly alter the organization, dosage, and function of multiple genes, contributing to a diverse range of human diseases. Based on their genomic and clinical consequences, SVs are categorized as balanced or unbalanced. Balanced rearrangements, such as translocations and inversions, which disrupt the organization and orientation of the DNA sequence without a net loss or gain of genetic material, and are generally asymptomatic in carriers. In contrast, unbalanced rearrangements, including deletions, duplications, and insertions, lead to changes in DNA dosage and are known as copy number variants (CNVs), often associated with clinically abnormal phenotypes.^[2]^

In recent years, CNVs have been implicated in a broad spectrum of human diseases, ranging from common neurodevelopmental and psychiatric disorders such as intellectual disability, autism spectrum disorders, and schizophrenia, to rare dysmorphic and malformative syndromes.^[3,4]^ Among CNVs, large duplications encompassing multiple genes can result in partial trisomies, which are typically associated with global developmental delay, intellectual disability, distinctive dysmorphic features, and congenital anomalies.^[5,6]^ These partial trisomies often arise from unbalanced chromosomal rearrangements, which are frequently inherited from phenotypically normal parents who carry balanced translocations.

In this report, we describe a rare case of a female infant presenting with 2 distinct pathogenic duplications involving chromosomes 9p24.3-q21.13 and 12p13.33-p12.1. Both duplications were identified using whole-genome CNV analysis and subsequently determined to result from an unbalanced segregation of a maternally inherited balanced translocation involving chromosomes 9 and 12. To our knowledge, this represents one of the few documented cases of concurrent pathogenic duplications involving these 2 chromosomal regions, contributing valuable insight into the phenotypic spectrum associated with complex chromosomal rearrangements. This case report follows the CARE Guidelines.^[7]^

## 2. Materials and methods

### 2.1. Materials

Peripheral venous blood samples were collected from the proband and both parents in heparinized and ethylenediaminetetraacetic acid tubes for conventional karyotyping and molecular CNV analysis. Written informed consent was obtained from the patient’s parents for publication of this case report and any accompanying clinical details and images, and the study was approved by the Biomedical Research Ethics Committee, Umm Al-Qura University (approval number: HAPO-02-K-012-2025-06-2822).

### 2.2. Karyotyping

Conventional karyotyping was performed on peripheral blood lymphocytes using standard G-banding techniques. A total of 20 metaphase spreads were analyzed, with a banding resolution of approximately 550 bands. Chromosome analysis was carried out according to standard laboratory protocols.

### 2.3. Genome-based CNV analysis

Genomic DNA was extracted from peripheral blood lymphocytes using standard protocols. Direct sequencing was performed using paired-end 2 × 150 bp reads on an Illumina next-generation sequencing platform, achieving a mean coverage of approximately 10× across the target region. Sequence reads were aligned to the human reference genome (GRCh37/hg19), and CNVs were identified using NxClinical software (version 5.1; BioDiscovery). CNVs meeting internal quality control criteria were subsequently confirmed using alternative methods. Genomic coordinates are reported according to the GRCh37/hg19 reference assembly.

### 2.4. Timeline

- March 5, 2020: 3rd pregnancy achieved with abnormal fetal ultrasound.
- August 5, 2020: The proband was born as a female infant with dysmorphic features and multiple congenital malformations.
- October 1, 2020: Conventional karyotype was conducted.
- April 26, 2021: Genome sequencing-based CNV analysis was conducted followed by parental karyotype analysis.
- January 1, 2022: The proband passed away.

### 2.5. Patient information

**Figure 1.**
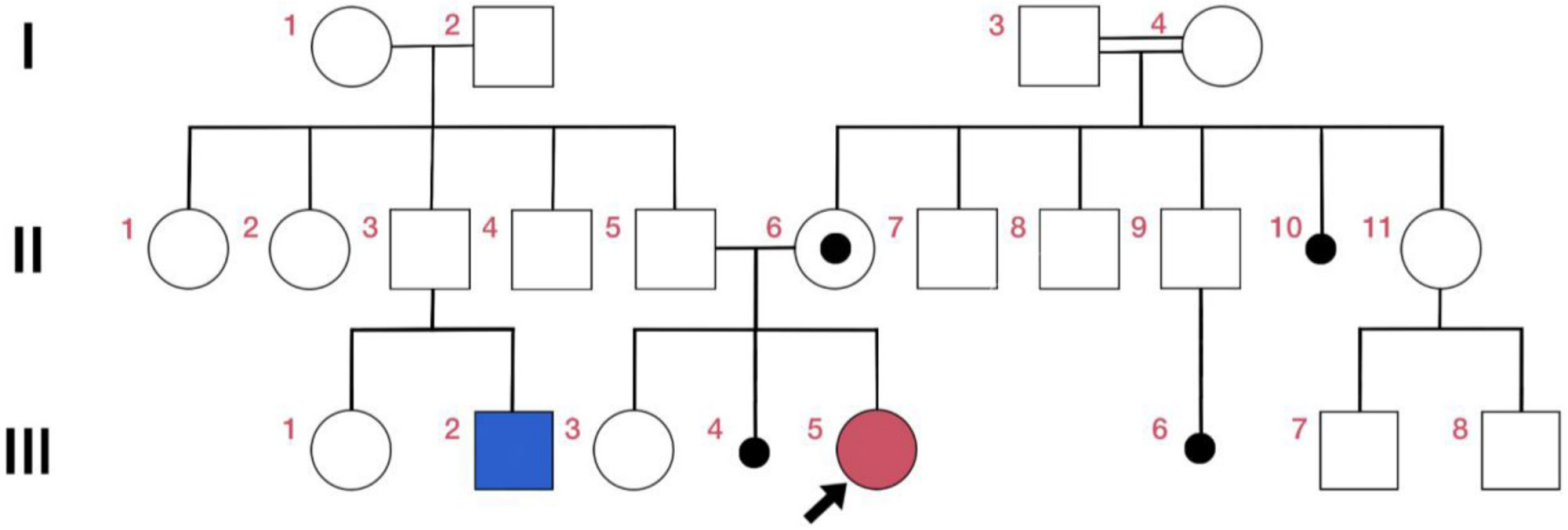
Pedigree of the proband’s family. Individual III-2: affected child with trisomy of chromosome 21. Individual III-5: the proband (black arrow) with concurrent partial trisomies of chromosomes 9p and 12p, described as 47,XX,+der(9)t(9;12)(q21.13;p12.1). Individual II-6: mother of the proband, a carrier of a balanced translocation, 46,XX,t(9;12)(q13;p11.2).

The case presented in this study was a baby girl born to 2 healthy, non-consanguineous parents (father, 31 years old; mother, 33 years old) who had 3 spontaneous pregnancies (Fig. 1). The first pregnancy was uncomplicated, resulting in the birth of a healthy girl via cesarean section due to postdate gestation. The second pregnancy ended in an early miscarriage at 4 weeks of gestation. The third pregnancy, which is the subject of this report, resulted in a baby girl who was delivered via cesarean section at term. This pregnancy was marked by the presence of intrauterine growth restriction, and reduced fetal movements were found on antenatal ultrasound screening.

### 2.6. Clinical findings

**Figure 2.**
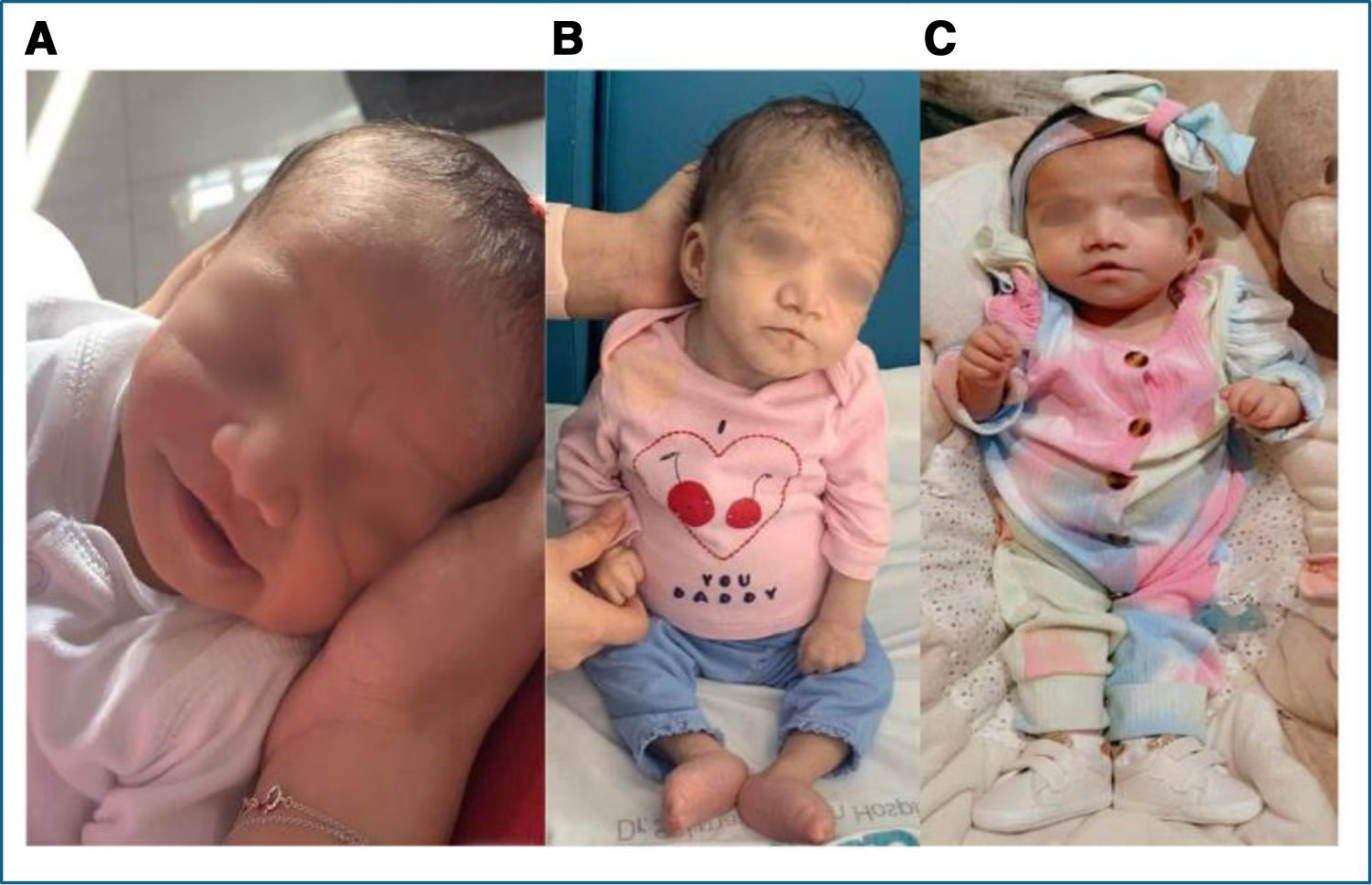
Craniofacial features of the proband, at birth (A), at the age of 4 months (B), and at the age of 12 months (C).

At birth, the baby had normal Apgar scores, and growth parameters were below the third centile of the standard growth chart of a normal newborn. In addition, she exhibited multiple dysmorphic features and congenital anomalies, including hypertelorism, low-set ears, fisted hands, talipes equinovarus (clubfoot), imperforate anus, and left hip dislocation (Fig. 2A). A comprehensive neonatal evaluation, including an echocardiogram, brain and abdominal ultrasounds, an eye examination, and hearing assessments, was performed at birth, with no abnormal findings reported. However, despite the initial normal assessment, the child displayed significant clinical issues during her first year of life. She was found, at the age of 12 months, to have global developmental delay as she was unable to maintain her head (head lag), sit independently, or crawl. Moreover, she showed generalized hypotonia, recurrent pneumonia, and failure to thrive. Craniofacial features became more prominent in the form of microcephaly, a large, prominent forehead, hypertelorism, a narrow nasal tip, a short philtrum, a thin upper lip, an inverted lower lip, and downturned corners of the mouth (Fig. 2B, C).

### 2.7. Diagnostic assessment

Given the presence of multiple congenital anomalies and dysmorphic features, conventional karyotyping was performed at the age of 2 months to identify any gross chromosomal aberrations. Samples of venous blood were collected and sent for cytogenetic analysis, which revealed an additional structurally abnormal chromosome 9 with a terminal deletion at band q22.2, described as 47,XX,+del(9)(q22.2), suggesting a preliminary diagnosis of partial trisomy 9p.

**Figure 3.**
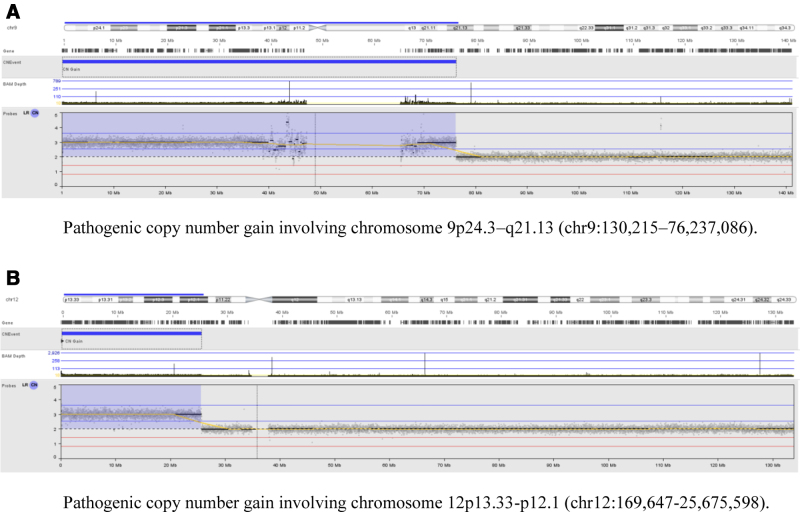
Genome sequencing-based CNV analysis profile identifying 2 pathogenic duplications in the proband. (A) An approximately 76.1 Mb terminal copy number gain of chromosome 9p24.3-q21.13 (chr9: 130,215–76,237,086). This region encompasses 195 OMIM genes, starting with *CBWD1* (OMIM #611078) and ending with *ANXA1* (OMIM #151690), and results in a partial trisomy of chromosome 9p. (B) An approximately 25.5 Mb terminal copy number gain of chromosome 12p13.33-p12.1 (chr12: 169,647–25,675,598), resulting in partial trisomy 12p. This region encompasses 218 OMIM genes, starting from *IQSEC3* (OMIM #612118) to *LMNTD1* (OMIM #617254). CNV = copy number variant, OMIM = Online Mendelian Inheritance in Man.

To further delineate the chromosomal imbalances, genome sequencing-based CNV analysis was subsequently performed. This analysis identified a pathogenic 76.1 Mb duplication spanning chromosome 9p24.3-q21.13, consistent with the initial karyotype finding. In addition, a second pathogenic CNV was detected, comprising a 25.5 Mb duplication of chromosome 12p13.33-p12.1 (Fig. 3). These results confirmed the presence of 2 partial trisomies involving chromosomes 9p and 12p.

**Figure 4.**
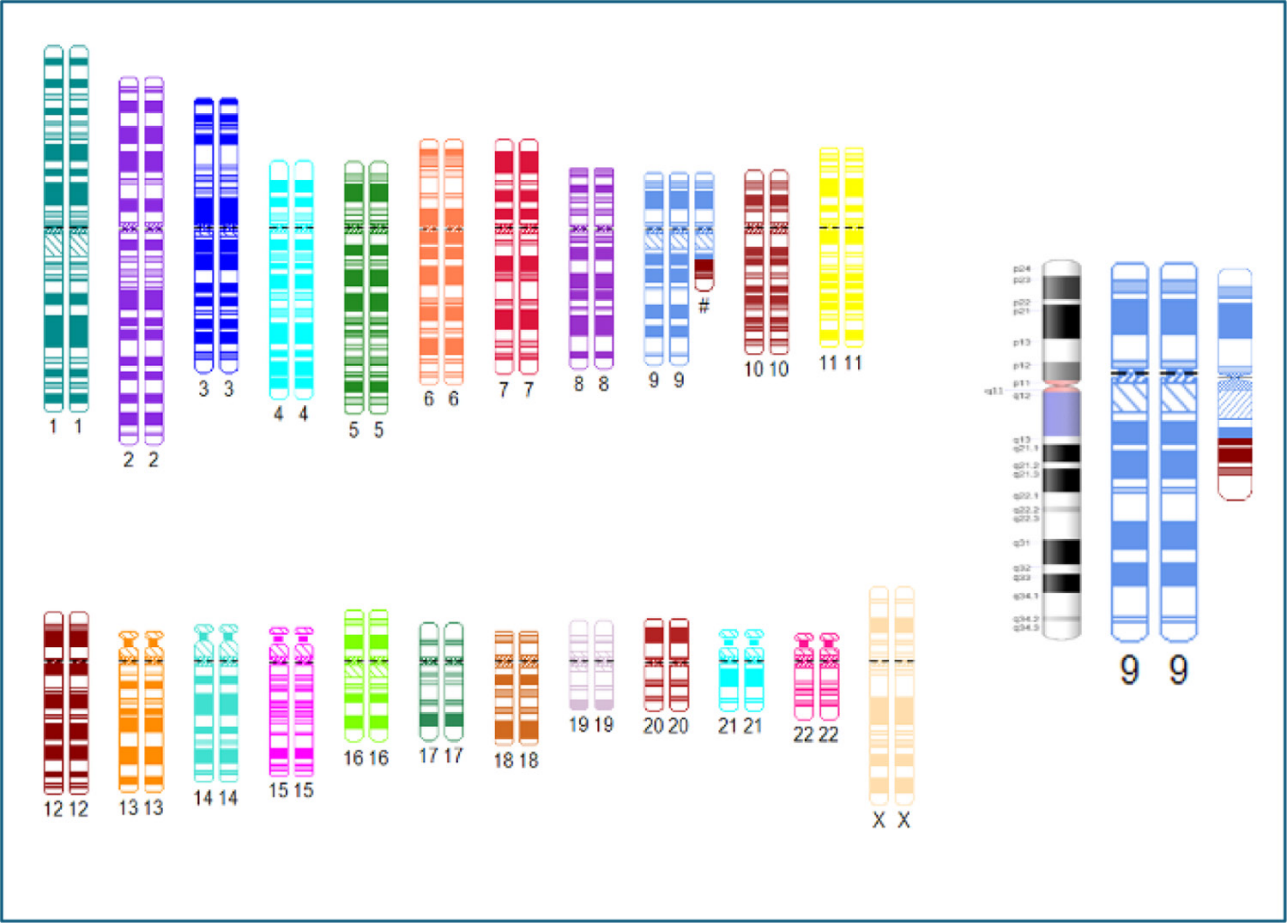
Ideogram of the proband’s karyotype showing an extra copy of chromosome 9 (#), which is redefined after genome sequencing-based CNV analysis, as a derivative chromosome 9 with a translocation between chromosomes 9 and 12, described as 47,XX,+der(9)t(9;12)(q21.13;p12.1). CNV = copy number variant.

These findings suggest that the additional chromosome 9 material found in this infant’s karyotype was, in fact, a derivative chromosome 9 resulting from an unbalanced translocation between chromosomes 9 and 12, described as der(9)t(9;12)(q21.13;p12.1), rather than a single duplication as initially presumed. The karyogram was not available from the testing laboratory; therefore, an ideogram is provided to illustrate the identified chromosomal abnormality (Fig. 4). The ideogram was generated using the CyDAS Online Analysis Site.^[8]^

**Figure 5.**
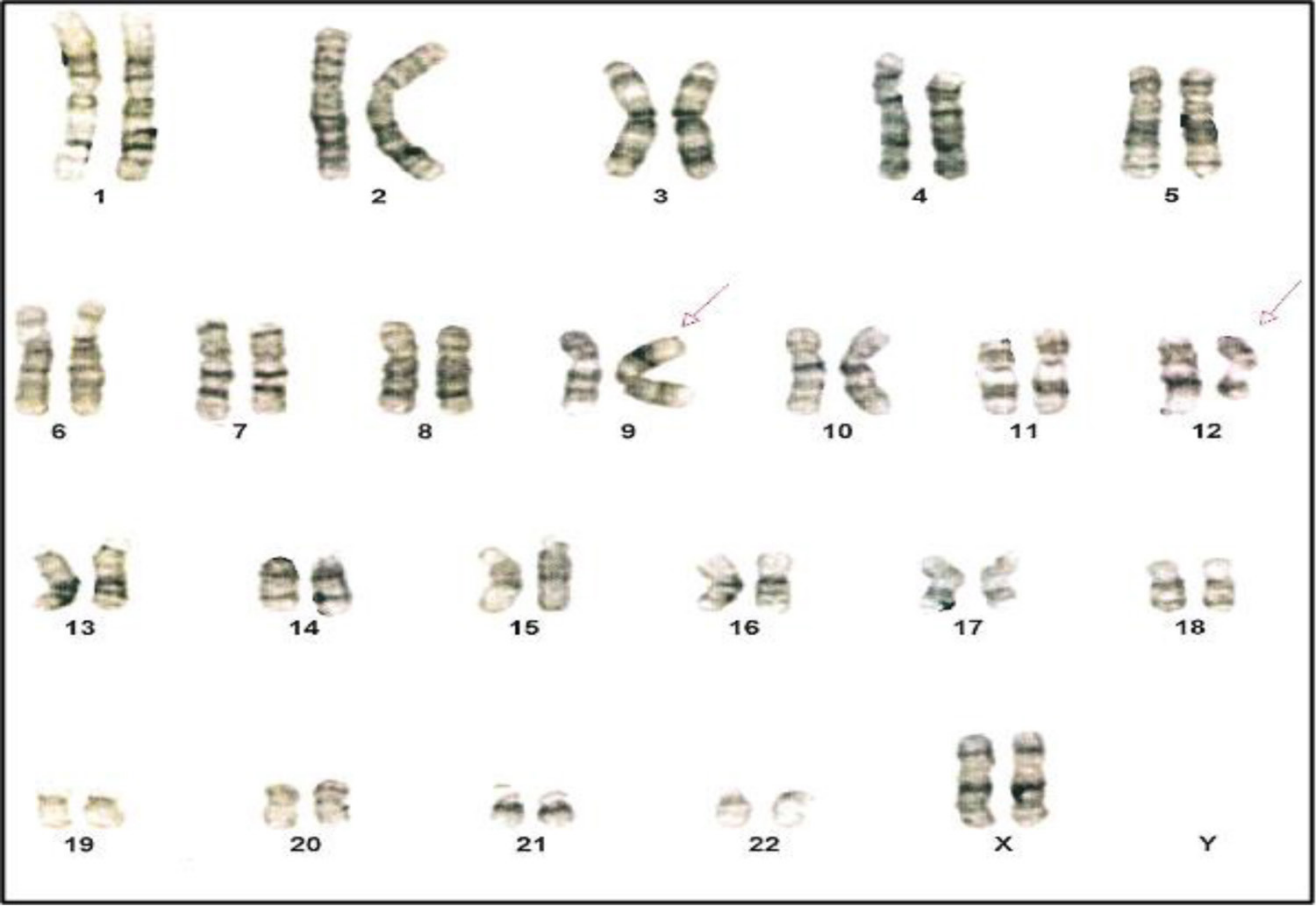
Maternal karyotype showing a balanced translocation between chromosomes 9 and 12 described as 46,XX,t(9;12)(q13;p11.2).

Parental cytogenetic analysis was subsequently undertaken to determine the origin of this rearrangement. The father’s karyotype was normal (46,XY). However, the mother was identified as a balanced translocation carrier, with a karyotype of 46,XX,t(9;12)(q13;p11.2; Fig. 5). The observed discrepancy in breakpoint localization between the infant and the mother is most likely attributable to differences in the methods’ resolution. No cytogenetic testing was performed in other family members due to unavailability of samples.

### 2.8. Follow-up and outcomes

At the age of 12 months, the infant developed a severe respiratory infection. Despite supportive care, her condition deteriorated, and she passed away a few weeks later.

## 3. Discussion

Partial trisomy of the short arm of chromosome 9 (9p) is one of the more frequently reported autosomal partial trisomies, with over 150 cases documented in the literature. Despite variations in duplication sizes, chromosomal breakpoints, and the coexistence of additional CNVs, affected individuals consistently exhibit characteristic dysmorphic features. These include microcephaly, downslanting palpebral fissures, hypertelorism, a prominent nose, a short philtrum, downturned corners of the mouth, and low-set ears.^[9]^ Additional commonly reported clinical features encompass developmental delay, short stature, digital anomalies, and intellectual disability.^[10,11]^ According to the UCSC Genome Browser (https://genome.ucsc.edu/), the duplicated region in the present case (chr9: 130,215–76,237,086) encompasses multiple dosage-sensitive and disease-associated genes whose increased copy number may disrupt neurodevelopment, skeletal growth, and morphogenesis. Among these, *FREM1* encodes an extracellular matrix protein expressed in epithelial basement membranes during embryogenesis; biallelic loss-of-function variants are associated with autosomal recessive malformative syndromes characterized by craniofacial anomalies, renal agenesis, and anorectal malformations.^[12]^ The clinical features observed in our patient – including craniofacial dysmorphism, microcephaly, and imperforate anus – partially overlap with this spectrum, although renal anomalies were absent. *DOCK8*, a guanine nucleotide exchange factor involved in immune signaling and cytoskeletal regulation, is implicated in autosomal recessive combined immunodeficiency^[13]^; the recurrent chest infections in our patient may reflect dosage-related effects involving this gene. In addition, *SMARCA2*, which encodes an ATP-dependent chromatin-remodeling protein, is associated with Nicolaides–Baraitser syndrome, characterized by severely impaired intellectual development, early-onset seizures, and dysmorphic facial features,^[14]^ and may contribute to the observed growth retardation in the present case. In addition, *RFX3*, *PTPRD*, and *VLDLR* are genes with important roles in neurodevelopment and neuronal function. *RFX3* encodes a transcription factor involved in ciliary gene regulation and brain development, with monoallelic variants associated with autism spectrum disorder and other neurodevelopmental phenotypes.^[15]^ *PTPRD* encodes a receptor-type protein tyrosine phosphatase that plays a key role in neuronal signaling, synaptic organization, and brain development, and its dysregulation has been linked to cognitive impairment and neurobehavioral disorders.^[16]^ *VLDLR*, a component of the Reelin signaling pathway, is essential for neuronal migration and cerebellar development; biallelic loss-of-function variants are associated with cerebellar hypoplasia, intellectual disability, and ataxia.^[17]^ According to the Online Mendelian Inheritance in Man database (https://www.omim.org/) and ClinGen databases (https://www.clinicalgenome.org/), these genes are dosage-sensitive and typically associated with loss-of-function haploinsufficiency pathogenic variants. There is no sufficient evidence to support triplosensitivity. However, their presence in 3 copies in this patient, along with the concordance with the clinical phenotype of the present case and previous studies, suggests that potential overexpression may disrupt these genes’ functions and underlie the observed phenotype.

In contrast, duplications of the short arm of chromosome 12 (12p) are considerably rarer, with approximately 50 cases described to date.^[18]^ The phenotypic variability observed in 12p duplication syndromes largely depends on the size of the duplicated segment and the presence of additional chromosomal rearrangements.^[19]^ Pure 12p duplications are typically associated with developmental delay, intellectual disability, hypotonia, and a distinct craniofacial phenotype that includes a high forehead, short nose with anteverted nostrils, flat and round facial contours, deep-set eyes, low-set ears, and a long philtrum.^[19,20]^ Izumi et al proposed that increased dosage of genes within the 12p13.31 region may be sufficient to produce the core phenotype observed in 12p duplication and Pallister–Killian syndrome. In particular, *ING4*, *CHD4*, and *MFAP5* have been highlighted as strong candidate genes due to their roles in cell proliferation and differentiation and their association with neurological disorders.^[21]^ Furthermore, duplications involving genes such as *GRIN2B*, *SOX5*, *SCN8A*, and *PIANP* within the 12p12.1-p13.3 regions have been found in individuals with developmental delay. These genes are critically involved in central nervous system and craniofacial development, supporting their role in the neurological manifestations observed in patients with 12p duplication.^[20]^ The case presented here demonstrated overlapping clinical features of both 9p and 12p duplications, notably developmental delay, growth retardation, and hypotonia. However, the craniofacial features observed in our patient more closely resembled those classically associated with partial trisomy 9p.

The coexistence of duplications involving both 9p and 12p in a single individual is exceptionally rare. To date, only a limited number of cases have been reported. Recently, Ming et al described a neonate with concurrent partial trisomies of 9p and 12p, presenting with growth retardation, hypertonia, absent head control, congenital heart defects including atrial septal defect, patent ductus arteriosus, and tricuspid valve insufficiency, and characteristic craniofacial features such as a prominent forehead, widely spaced eyes, low-set ears, short philtrum, thin upper lip, downturned corners of the mouth, clenched fists, and ankle valgus deformities.^[22]^ The phenotypic presentation of our case closely mirrors that of the reported patient, except for congenital heart disease, which was absent in the present patient. This observation may reflect phenotypic variability within this exceedingly rare genomic disorder. Further documentation and analysis of additional cases with similar SVs are essential to better define the phenotypic spectrum and clinical implications associated with concurrent 9p and 12p duplications.

Partial trisomies can be identified by karyotype analysis as duplications, supernumerary marker chromosomes, or derivative chromosomes involving segments from another chromosome.^[19,20]^ Approximately 60% to 70% of unbalanced chromosomal rearrangements are inherited from a parent carrying a balanced translocation, with a predominance of maternal origin.^[23,24]^ Although carriers are typically phenotypically normal, abnormal meiotic segregation can result in gametes with unbalanced chromosomal content. In the present case, the coexistence of duplications involving chromosomes 9p and 12p is most consistent with an unbalanced segregation event arising from a maternal balanced translocation, likely via a 3:1 segregation mechanism.

CNV analysis is currently regarded as the first-line genomic investigation for individuals presenting with unexplained global developmental delay, intellectual disability, autism spectrum disorders, and multiple congenital anomalies.^[25]^ The detection of CNVs depends largely on the variant size and the resolution of the technique used. Conventional karyotyping is capable of identifying large, microscopic CNVs typically greater than 5 megabases in size, while smaller, submicroscopic CNVs require molecular-based technologies such as comparative genomic hybridization arrays or whole-genome sequencing (WGS)-based techniques for detection. More recently, WGS-based CNV analysis has emerged as a powerful clinical diagnostic tool for identifying pathogenic CNVs across a wide range of neurodevelopmental and dysmorphic malformative disorders, demonstrating a superior diagnostic yield compared with traditional array-based platforms.^[26–28]^ To our knowledge, this report represents the first documented case of concurrent 9p and 12p duplications in a dysmorphic neonate identified through WGS-based CNV analysis. This case highlights the value of WGS-CNV analysis in uncovering genomic alterations that may be missed by conventional methods and underscores its utility in refining genotype–phenotype correlations in rare structural genomic disorders.

## 4. Conclusion

We describe the phenotypic and genomic features of a rare case of concurrent partial trisomies 9p and 12p arising from a maternal balanced translocation. This case highlights the importance of integrated cytogenetic and genomic analyses to accurately characterize complex chromosomal abnormalities. It also underscores the critical role of parental cytogenetic evaluation in determining recurrence risk and guiding genetic counseling and reproductive planning.

## Acknowledgments

We thank PerkinElmer Genomics and GenaT Laboratories for conducting the molecular and cytogenetic analyses in this study. The authors used ChatGPT (GPT-5, OpenAI) solely for language editing and formatting support. All scientific content and interpretations are the responsibility of the authors.

## Author contributions

**Data curation:** Zohor Azher, Malak Alsabban, Lujain Akram Ali.

**Formal analysis:** Zohor Azher.

**Investigation:** Zohor Azher.

**Methodology:** Zohor Azher.

**Project administration:** Zohor Azher.

**Writing – original draft:** Zohor Azher, Malak Alsabban, Lujain Akram Ali.

**Writing– review & editing:** Zohor Azher, Hind Naffadi, Samar Ekram, Rami Obaid.
